# A Phase I Study of High-Dose Calcitriol in Combination with Temozolomide for Patients with Metastatic Melanoma

**DOI:** 10.3390/jpm4040448

**Published:** 2014-10-17

**Authors:** Erin Pettijohn, Brenda Martone, Alfred Rademaker, Timothy Kuzel

**Affiliations:** 1 Division of Hematology/Oncology, Northwestern University, 676 N. St Clair, Suite 850, Chicago, IL 60611, USA; E-Mails: erin-pettijohn@northwestern.edu (E.P.); bmartone@nmff.org (B.M.); 2 Department of Medicine, Northwestern University, 251 E. Huron, Galter Suite 3-150, Chicago, IL 60611, USA; 3 Division of Biostatistics, Department of Preventative Health, Feinberg School of Medicine, Northwestern University, 680 N. Lakeshore Drive, Suite 1400, Chicago, IL 60611, USA; E-Mail: rademaker@northwestern.edu

**Keywords:** metastatic melanoma, vitamin D receptor polymorphisms, temozolomide, calcitriol, Taq1, Fok1

## Abstract

*Background*: Temozolomide is efficacious as an oral alternative for patients with metastatic melanoma (MM). Calcitriol has anti-proliferative properties and vitamin D receptor (VDR) polymorphisms are associated with alterations in melanoma susceptibility and progression. *Methods*: Tem 150 mg/m^2^ was administered on days 2–8 and 16–22 every 28 days. Calcitriol was given on days 1 and 15 every 28 days. VDR gene analysis was completed using PCR-RFLP based assays. Tolerability was the primary objective with secondary objectives of time to progression (TTP) and overall survival (OS). *Results*: Twenty pts with MM were registered. Cytopenias and thrombosis were the most common grade 3 or 4 toxicities. Median TTP was 1.8 mo. Pts with high-risk VDR genotype tt+/−ff (*n* = 6) had an OS of 3.8 mo from time of enrollment, compared to 7.4 mo for those with non-tt/ff genotypes (*n* = 11), although not statistically significant (HR = 1.20, 95% CI 0.41–3.53, *p* = 0.74). *Conclusions*: The extended dosing of Tem with calcitriol is a well-tolerated regimen. The trend toward improved OS in non-tt/ff VDR genotypes is consistent with prior studies associating the tt/ff genotype with biologic aggressiveness.

## 1. Introduction

Metastatic melanoma (MM) is relatively resistant to systemic chemotherapy and carries a dismal prognosis in the setting of disseminated disease with a median survival of 8.9 months [1]. Various other treatment approaches have been evaluated, such as the immunotherapies, high-dose Interleukin-2 and ipilimumab, and the targeted small molecule braf and mek inhibitors. Although recently ipilimumab, vemurafenib, debrafenib and tremetinib have demonstrated a modest improvement in progression-free survival (PFS) and overall survival (OS) in controlled trials relative to the use of chemotherapy, the majority of patients are not cured [2,3]. Furthermore, the use of vemurafenib, debrafenib and tremetinib are restricted to patients with activating mutations in the BRAF gene. For patients who are intolerant to, relapse, or are not candidates for the above first line therapies, systemic chemotherapy may still be indicated. Single agent dacarbazine (DTIC) is the only cytotoxic agent approved by the FDA for the treatment of metastatic melanoma, achieving response rates as high as 15%–20%. However, the responses lack durability [1]. Response rates may be higher with combination therapy, but there is no significant increase in time to progression or survival [4].

Temozolomide is an oral alkylating agent with a broad spectrum of anti-tumor activity and relatively modest toxicity. A randomized trial of temozolomide *versus* DTIC in Stage IV melanoma showed a longer progression free survival in the temozolomide group (1.9 months *versus* 1.5 months; *p* = 0.12), but overall survival and response rates were no better [5]. No major differences in drug safety were observed. The traditional dosing schedule of temozolomide is 200 mg/m^2^ orally for 5 consecutive days every 28 days. However, temozolomide dosed at 150 mg/m^2^ daily for 7 days every other week permits a 2.1-fold greater drug exposure than the conventional schedule, saturates resistance mechanisms, and has had favorable activity when investigated in patients with glioblastoma, both in the neo-adjuvant setting and as maintenance therapy [6,7]. Patel *et al.* studied the seven-day on/seven-day off temozolomide dosing schedule *versus* DTIC in stage IV melanoma, finding a higher overall response rate with similar progression free survival in the temozolomide arm, but with a shorter duration of response and worse toxicity profile [8].

Calcitriol (1,25-dihydroxyvitamin D3) is the most active metabolite of vitamin D, and is a well-known potent regulator of cell growth and differentiation [9,10]. Preclinical studies have demonstrated that several melanoma cell lines express the vitamin D receptor (VDR), and that 1,25-dihydroxy-vitamin D3 has anti-proliferative and pro-differentiation effects in cultured melanoma cells and in melanoma xenografts [11]. Mason *et al.* confirmed that multiple melanoma cell lines expressed the VDR, and treatment with calcitriol induced differentiation [12]. In a pre-clinical study focusing on melanoma metastases, Yudoh *et al.* demonstrated that calcitriol could inhibit *in vitro* the invasiveness in an extracellular matrix, and *in vivo* inhibit the development of pulmonary metastases from the B16 cell line in a mouse model [13]. Synergistic or additive effects of calcitriol with cytotoxic chemotherapy were reported in the ASCENT-1 trial of calcitriol plus docetaxel in androgen-independent prostate cancer, with significant improvement in tumor response, skeletal event-free survival time, and frequency of serious adverse events [14]. These results were not replicated in the phase III ASCENT-2 trial, although docetaxel dosing also differed between the two study arms in that study [15].

In addition, certain polymorphisms of the VDR in humans have been associated with both melanoma susceptibility and Breslow thickness. Hutchinson *et al.* studied the VDR Taq1 and Fok1 polymorphisms and found that the combined variant tt/ff genotype (Taq1 and Fok1) was associated with tumors thicker than 3.5 mm (OR = 31.5, *p* = 0.001) [16], while homozygosity for the wild-type allele of the Fok1 (FF) correlated with a reduced melanoma risk. Significant associations have also been found between the Bsm1 bb genotype and Breslow thickness by Santonocito *et al.* [17]. Other VDR polymorphisms implicated but less robustly studied include EcoRV, which has been shown to be associated with presence of distant metastases and thicker Breslow measurements as well as Cdx2 which was not found to have a significant association with melanoma risk or outcome in one small study [18,19]. Given the favorable effects of calcitriol on chemotherapy toxicity noted in the ASCENT-1 trial [14] (not confirmed in ASCENT II, but the different docetaxel dosing in the arms makes this comparison less clear), and the effect *in vitro* on VDR expressing melanoma cell lines, we hypothesized that the combination of calcitriol plus temozolomide might result in enhanced response rates and a more tolerable side effect profile.

## 2. Experimental 

### 2.1. Patient Eligibility/Selection

This prospective non-randomized phase Ib study was conducted from January 2006 through April 2012. All patients provided written informed consent approved by the Northwestern University Institutional Review Board, and dose escalation was overseen by the data safety monitoring board of the Robert H. Lurie Comprehensive Cancer Center. Main inclusion criteria were age ≥18 years with histologically confirmed stage IV metastatic malignant melanoma from any primary site with measurable disease. Patients who had at least one prior systemic therapy (aside from prior temozolomide or dacarbazine) were eligible, as well as those with no prior therapy but who were not candidates for high-dose interleukin-2. Patients must not have received radiotherapy, chemotherapy or immunotherapy in the 4 weeks prior to the first study treatment. EGOG performance status of 0, 1 or 2 was required, with baseline laboratory function as follows: creatinine <2.0 mg/dL, calcium <10.5 mg/dL, phosphorus <4.3 mg/dL, total bilirubin within institutional normal range, platelets >100,000 per mm^3^, and white blood cell count >3500 per mm^3^.

### 2.2. Study Design

The primary objective was to assess the safety and tolerability of the seven-day on/seven-day off temozolomide dosing schedule in combination with calcitriol dose escalation in patients with metastatic melanoma. Secondary objectives included tumor response, time to progression, and correlation of overall survival with VDR gene polymorphisms. Temozolomide was given at a dose of 150 mg/m^2^ orally on days 2–8 and 16–22 on a 28-day cycle until progression of disease or significant toxicity up to a maximum of 12 cycles. A standard 3 + 3 scheme was employed for administration of the calcitriol, beginning with lower doses in order to avoid unexpected toxicity when combined with temozolomide. Calcitriol in capsule form was given on days 1 and 15 every 28 days with 0.2 mcg/kg given to the first 3 patients (cohort 1), 0.3 mcg/kg to the second 3 patients (cohort 2), and 0.5 mcg/kg to the next 3 patients (cohort 3). The remaining patients received maximally tolerated doses of calcitriol, which was determined to be 0.5 mcg/kg given the favorable side-effect profile (detailed below). Saturated absorption of calcitriol has been found to be achieved at this dose with higher dosing not resulting in increased exposure [20]. Therefore the trial was designed not to have any dose escalation beyond the 0.5 mcg/kg dose. All toxicity was graded using the National Cancer Institute Common Toxicity Criteria (NCI CTC), version 3.0. Hematologic toxicity was evaluated with complete blood counts (CBC) within 72 h of scheduled dosing. Temozolomide dose delays and adjustments occurred if the absolute neutrophil count (ANC) was <1500/mm^3^ or platelet count <100,000/mm^3^. Temozolomide could then be given with dose adjustment to 120 mg/m^2^ for a nadir ANC of 500–999/mm^3^ or nadir platelet count of 25,000–49,999/mm^3^, or 100 mg/m^2^ for nadir ANC <500/mm^3^ or platelets <25,000/mm^3^. If after 3 weeks, the ANC remained <1500/mm^3^ or platelets <100,000/mm^3^, the patient was withdrawn from the study and the delay in dosing reported as a serious adverse event. For non-hematologic study drug related toxicity, dose reduction to 120 mg/m^2^ for grade 3 toxicity was employed, with discontinuation of therapy and removal from study for grade 4 toxicity. There were no dose modifications for calcitriol. Patients were pre-medicated with 5HT3 antagonists to decrease nausea and vomiting from temozolomide.

### 2.3. Patient Evaluation

All baseline evaluations were performed within four weeks prior to the beginning of treatment, including complete history, physical exam, tumor measurements, CBC with differential, comprehensive chemistry, calcium, albumin, phosphorus, and performance status. The same method of assessment was used at baseline as during follow-up. Response and progression was evaluated using the Response Evaluation Criteria in Solid Tumors (RECIST) [21]. Patients must have completed one treatment cycle to be evaluable for response, with planned imaging as applicable just prior to week 8 to assess disease burden. Complete response (CR), partial response (PR), progressive disease (PD), and stable disease (SD) were defined by RECIST criteria. To be assigned a status of PR or CR, changes in tumor measurements must have been confirmed by repeat assessments performed no less than four weeks after the criteria for response was first met. In the case of SD, follow-up measurements must have met the SD criteria at least once after study entry at a minimum interval of four weeks. Time to progression (TTP) was calculated from the initiation of therapy to the date of documented disease progression. Adverse events were assessed on day one of each subsequent cycle of therapy and/or at time of progression.

### 2.4. Vitamin D Receptor SNP Genotyping 

Peripheral blood was drawn for VDR gene analysis using PCR-RFLP based assays prior to initiation of the first cycle of treatment. Primers to amplify the regions containing each SNP of interest were designed using Pyromark Assay Design Software (version 2.0.1.15, Qiagen, Hilden, Germany, 2008). In each case, one primer was biotinylated to allow for purification of the DNA strand for analysis by pyrosequencing. PCR was performed using PCR Master Mix from Promega (Madison, WI, USA). For each primer set, PCR cycling conditions were as follows: an initial 5 min denaturation at 95 °C followed by 50 cycles of PCR with 30 s each of denaturation (95 °C), annealing (58 °C), and extension (72 °C). A final 10 min extension at 72 °C was included. PCR products were prepared for pyrosequencing in duplicate by binding the DNA to streptavidin-coated sepharose beads (GE Healthcare, Pittsburgh, PA, USA), and then using the Vacuum Prep Workstation (Qiagen, Valencia, CA, USA). Once on the vacuum prep tool, the DNA/beads were rinsed in 70% ethanol, denatured in 0.2 M NaOH, then washed in 10 mM Tris-acetate buffer (pH 7.6). The DNA/beads were then released into a pyrosequencing plate containing the appropriate sequencing primer. The plate was heated to 80 °C for 2 min, then cooled to room temperature, after which pyrosequencing commenced using PyroGold reagents on a Pyromark MD machine (Qiagen) according to the instructions of the manufacturer. Genotypes were resolved on the basis of peak height measurements using PyroMark MD software (version 1.0, Biotage AB, Uppsala, Sweden). Negative and positive control PCR reactions were included for every pyrosequencing run.

### 2.5. Statistical Analysis

Data are reported as frequencies, medians and ranges. Median and interquartile range (IQR) for overall survival and progression free survival are estimated using Kaplan-Meier curves. Subgroups are compared using the log-rank test and proportional hazards regression was used to calculate the hazard ratio.

## 3. Results

### 3.1. Patient Characteristics 

Twenty patients with a median age of 58 were enrolled between January 2006 and April 2012 (Table 1). All patients had confirmed measurable metastatic melanoma. Seventy-five percent were males, and median ECOG performance score was one. All patients were assessable for response and toxicity. The median follow-up from the study enrollment date was 5.5 months (range 0.5–77.1 months). 

**Table 1:** Baseline Patient Characteristics.

Characteristic	*n* = 20
Median Age (range)	58 (30–86)
Race,* n* (%)	
Caucasian	20 (100)
Gender, *n* (%)	
Male	15 (75)
Female	5 (25)
Median time from initial diagnosis, years (range)	2.4 (0.6–10)
ECOG performance status, *n* (%)	
0	9 (45)
1	10 (50)
2	1 (5)

### 3.2. Drug Exposure

A total of 52 treatment cycles of temozolomide plus calcitriol were administered with a median of 2 cycles and range of 0.5 to 10 cycles. Of the 16 patients who received more than one cycle, seven required dose reductions of temozolomide. All 20 patients received planned doses of calcitriol as described above.

### 3.3. Safety/Toxicity

The study regimen was well tolerated overall with hematologic toxicity as the most common adverse effect. The most common grade 3 or 4 toxicities were leukopenia, lymphopenia, anemia, thrombocytopenia, and thrombosis at 10% incidence each (Table 2). Grade 4 thrombocytopenia occurred in one patient after days 1–8 of treatment cycle one. The patient was admitted with a grade 3 deep venous thrombosis, grade 3 neurotoxicity which was possibly related to the study drug, and a grade 3 infection which was likely not related to the study drug. Treatment was held and the patient unfortunately expired with rapidly progressive disease within 30 days of treatment. One patient experienced a grade 3 hemorrhage, rash, and anorexia, in addition to grade 4 leukopenia and thrombocytopenia. The patient was taken off the study when evidence of progressive disease was noted after 1.5 cycles. An additional patient was found to have grade 1 thrombocytopenia that did not resolve after three weeks, and was therefore removed from the study. Notably, no serious infections related to the study drug or febrile interstitial pneumonitis were seen as reported in prior studies involving temozolamide [7,8]. Hypercalcemia was not noted in any patients receiving high-dose calcitriol. 

**Table 2:** Grade 3 or 4 Toxicities.

Toxicity	No.	%
Thrombocytopenia	2	10
Vascular	2	10
Nausea/vomiting	1	5
Leukopenia	2	10
Fatigue	1	5
Anemia	2	10
Lymphopenia	2	10
Hemorrhage	1	5
Rash	1	5
Anorexia	1	5

### 3.4. Efficacy Evaluation

**Figure 1:**
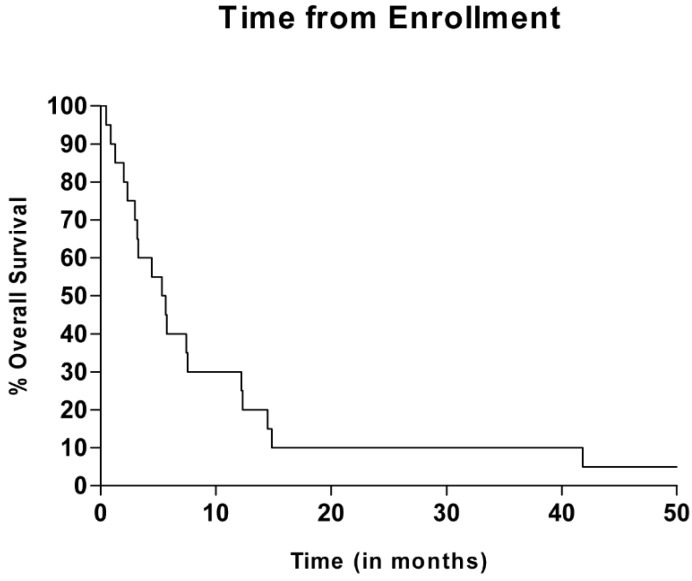
Overall Survival with Temozolomide and Calcitriol in Metastatic Melanoma.

The overall response rate was 10% with two patients achieving a partial response. The median TTP was 1.81 months (IQR = 1.15–1.99 months) with a mean of 2.7 cycles administered. The median OS was 5.5 months, IQR = 2.7–12.3 months, (Figure 1).

### 3.5. VDR Polymorphism Evaluation

**Figure 2:**
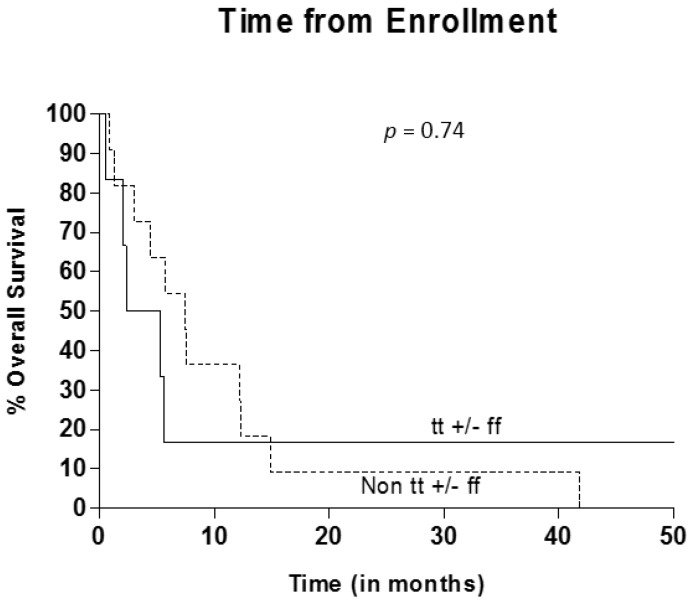
Overall Survival by vitamin D receptor (VDR) Genotype.

Vitamin D receptor gene analysis was completed on 17/20 patients evaluating for polymorphisms in Taq1, Fok1. Six patients were found to have tt or ff genotypes for the Taq1 and Fok1 genes. Patients with the VDR genotype (tt+/−ff), were found to have a trend for worse outcome with OS of 3.8 months (IQR =2.0–5.7 months) from time of enrollment, compared to 7.4 months (IQR = 3.0–12.3 months) for those with non-tt+/−ff genotypes (HR [tt+/−ff to non tt+/−ff] = 1.20, 95% CI 0.41–3.53, *p* = 0.74) although the difference was not statistically significant (Figure 2). 

## 4. Discussion

This study confirms the efficacy and tolerability of the seven-day on seven-day off temozolomide dosing schedule with addition of high-dose calcitriol in metastatic melanoma. In this limited phase Ib study, rates of hematologic toxicity were lower when compared to other studies using the same temozolomide dosing regimen. Clarke *et al.* reported grade 3 or 4 lymphopenia in 68% and leukopenia in 19% of patients in their temozolomide dose-dense arm [22]. Chinot *et al.* found 20% grade 3 or 4 thrombocytopenia and lymphopenia, 17% neutropenia, and 18% febrile interstitial pneumonitis associated with profound neutropenia or lymphopenia [7]. Patel *et al.* reported 45% grade 3 or 4 lymphopenia with otherwise similar rates of leukopenia, thrombocytopenia and anemia [8]. In our study, the most common grade 3 or 4 toxicities were leukopenia, lymphopenia, thrombocytopenia, anemia, and thrombosis with each occurring in only 10% of patients. High-dose calcitriol has been hypothesized in prior studies to decrease toxicity when given in combination with systemic chemotherapy. In the ASCENT-1 study comparing high-dose calcitriol plus docetaxel to placebo plus docetaxel in prostate cancer, there were fewer gastrointestinal (2.4% *vs.* 9.6%; *p* = 0.02) and thromboembolic (1.6% *vs.* 7.2%; *p* = 0.03) serious adverse events in the calcitriol arm [14]. In a follow-up phase III ASCENT-2 study comparing docetaxel plus high-dose calcitriol to docetaxel plus prednisone in prostate cancer, rates of febrile neutropenia were significantly lower in the calcitriol arm (1.0% *vs.* 4.6% control), although the authors argue that these differences are just as likely to be attributed to the fact that the calcitriol arm ultimately received less cumulative docetaxel therapy and therefore also experienced shorter survival times [15]. Although the number of patients in our study was small and toxicity is difficult to compare across studies, the improved safety profile of temozolomide given with calcitriol in our study compared to the previously reported toxicities of temozolomide raises the possibility of a protective effect of calcitriol, specifically in regards to myelosuppression, which deserves further investigation.

The TTP of 1.8 months with extended schedule dose temozolomide in our study was comparable to the TTP of 1.9 months with traditional dosing (200 mg/m^2^/day on days 1–5, repeated every 28 days) and 2.3 months with the previously studied extended dosing in metastatic melanoma by Patel *et al.* [5,8]. However, overall survival was significantly lower at 5.5 months compared to 9.1 months in the study by Patel, and 7.7 months with traditional dosing. Our study did allow previously treated patients to be included, which may have biased the results. Despite studies showing potential synergistic qualities of high dose calcitriol with cytotoxic chemotherapy, we were not able to demonstrate improved response rate or survival in this study.

Interestingly, in regard to VDR receptor polymorphisms in metastatic melanoma, we found a trend toward worse overall survival in those patients with the Taq1 and Fok1 (tt+/−ff) genotype compared to those with the other genotypes. Although this difference was not statistically significant in this small, early phase study, the results were consistent with previous studies showing increased biologic aggressiveness with regards to Breslow depth at time of diagnosis with the high-risk genotype (tt+/−ff). This is the only report we are aware of that attempted to correlate VDR polymorphisms with outcomes in metastatic melanoma patients. Although high-dose calcitriol did not appear to improve outcomes overall in this study, the question remains whether the increased risk of progression in the tt+/−ff subgroup is related to dysfunctional vitamin D regulation and could be attenuated by exogenous calcitriol. Alternatively, the high-risk VDR genotype may represent a biomarker for worse survival in metastatic melanoma, and thus represents an additional prognostic factor that should be controlled for in trials of advanced disease. Given the potential for prognostic significance in melanoma as well as a role as a possible future therapeutic target, the relationship between VDR polymorphisms and melanoma should be further investigated.
